# Bullying among students ecological insights from a school based adolescent health survey, Himachal Pradesh, India

**DOI:** 10.1371/journal.pone.0345468

**Published:** 2026-04-02

**Authors:** Deepika Bahl, Devika Mehra, Nikita Patel, Saroj Mohanty, Subha Sankar Das, Rishi Garg, Gaurav Sethi, Anjali Chauhan, Sunil Mehra

**Affiliations:** 1 Research and Programme, Mamta HIMC, Delhi, New Delhi, India; 2 Social Medicine and Global Health, Department of Clinical Sciences, Lund University, Malmö, Sweden; 3 Mamta HIMC, Delhi, New Delhi, India; 4 Research and Innovation, Mamta HIMC, Delhi, New Delhi, India; 5 Adolescent Health, Himachal Pradesh, India; University of Huelva: Universidad de Huelva, SPAIN

## Abstract

**Background and objective:**

Bullying among adolescents is a growing public health concern with serious mental, emotional, and social well-being consequences. This study aims to assess prevalence of physical and cyberbullying among school-going adolescents (13–17 years) in Himachal Pradesh, India, and identify risky and protective factors using an ecological framework descriptively.

**Methods:**

A cross-sectional, school-based survey was conducted across Himachal Pradesh using culturally adapted standardised questionnaire. Multivariate logistic regression analysis was performed to identify factors associated with bullying perpetration and victimization.

**Results:**

Out of 7563 adolescents surveyed, 18.41% reported involvement in physical or cyber bullying, with 13.96% involved in physical bullying and 9.64% in cyberbullying. Additionally, 15.60% adolescents reported being victims, including 11.40% experiencing physical and 7.88% cyberbullying.

*Associated Perpetration factors*:

**• Behavioral factors**: Increased risk was associated with junk food consumption, skipping breakfast, substance use, sexual activity, excessive screen time (>8 hours/day), and involvement in physical fights.

**• Emotional factors**: Feelings of hopelessness and nervousness were associated with increased odds of both physical and cyberbullying.

**• Family-related factors**: Adolescents with unskilled parents had higher odds of bullying others, while strong parental connectedness showed significant positive association.

*Victimization*

**• Demographic and behavioral factors**: Boys and adolescents from urban areas were more likely to be victims. Additional associated factors included unhealthy eating habits, substance use and sexual activity.

**• Emotional factors**: Feelings of disappointment and distress were associated with increased victimization

**• Family-related factors**: Parental connectedness was protective against both forms of bullying.

**Conclusion:**

Findings highlight significant prevalence of both physical and cyberbullying among adolescents in Himachal Pradesh, with distinct behavioral, emotional, and family-related risk factors. Parental connectedness proved protective, underscoring the need for integrated, multi-level interventions to foster healthier behaviors, emotional well-being, and safer schools. *Ayushman Bharat* School Health and Wellness Programme can be strengthened by tailoring its module based on the identified risk and protective factors.

## Introduction

Bullying among children and adolescents poses significant health risks and is increasingly recognised as a pervasive public health challenge [1]. Its manifestations can range from direct physical aggression or verbal abuse to indirect actions like social exclusion and online harassment (commonly referred as cyberbullying) [2]. A global meta-analysis published in 2025 estimates that approximately one in four children and adolescents have experienced bullying at least once, while around 16% have engaged in bullying behavior, and a similar proportion fall into the category of bully-victims (those who are both perpetrators and victims of bullying) [3]. In studies limited to India, estimates are more variable: victimization estimates from ~9% to ~80%, depending on definitions, region, and type of bullying [4].

Cyberbullying has become a growing concern in the digital realm [5]. Although it occurs less frequently than traditional bullying but there is a significant correlation between the two [6]. Globally, prevalence of cyberbullying preparation ranged from 6.0 to 46.3%, while victimization rates ranged from 13.99 to 57.5% [7]. Similarly, reported rates of cyberbullying victimization range from 3.3% to 30%, with some studies also highlighting the proportion of adolescents engaging in such behaviour [8].

Despite the high prevalence, bullying remain under prioritised in India’s national surveys including the National Family Health Survey [9], Comprehensive National Nutrition Survey [10], Global Adult Tobacco Survey [11], and Global Youth Tobacco Survey [12]. This gap highlights the urgent need for systematic monitoring and integration of bullying-related indicators into national adolescent health surveillance systems. Bullying has extensive physical and emotional consequences, not only for victims but also for perpetrators and bystanders [13–15]. Research has linked bullying to a range of mental health challenges, including depression, anxiety, self-harm, sleep disturbances, and reduced academic performance [16,17]. These adverse effects persist up to adulthood, with research studies showing that children who have been bullied had a 1.56 times higher risk of developing anxiety and a 1.80 times higher risk of developing depressive disorders compared to those who were not bullied [18]. Among adults who experienced bullying during childhood, the odds of smoking were 6.52 times higher and the odds for sexually transmitted diseases were 2.37 times higher in adulthood [19]. Such long term consequences underscores the need for deeper understanding of bullying dynamics during adolescence to inform the development of effective interventions [20].

It is crucial to consider the broader social context that shapes and sustains bullying behaviours. Bullying is not an isolated phenomenon but is influenced by interactions within a social network of individuals, their families, peer groups, society, community, and the broader culture [21]. Zastrow and Kirst-Ashman [22] provide an ecological framework that delineates influences of the microsystem, mesosystem, and macrosystem on these behaviours expressions.

The microsystem refers to the most immediate surroundings of an individual, incorporating their biological and psychological traits—such as age, gender, mental health, and personal behaviors. Evidence highlights adolescents who followed a healthy dietary pattern had a lower likelihood of engaging in bullying behavior (RR 0.67 [0.49, 0.92], whereas those with an unhealthy dietary pattern were more likely to be both perpetrators and victims of bullying (RR 1.29 [1.12, 1.48] [23,24]. Factors at this level can play an important role in bullying behaviour for example adolescents with poor self-esteem, exposure to violence, or emotional distress are more likely to engage in bullying or become victims [25].

Extending beyond, the mesosystem includes close social relationships, such as those with family, peers, and friends, which significantly shape the individual's daily experiences and social interactions. At this level, peer influence, family relationships, and social support systems play a major role. Adolescents who associate with aggressive peers or experience low parental connectedness are at the increased risk of bullying involvement [26]. At a broader level, the macrosystem encompasses community (school and neighbourhood) that form the larger context in which individuals live [21]). School climate, teacher responsiveness, and neighbourhood safety contribute significantly. Bullying is more common in schools with weak disciplinary systems, poor student-teacher relationships, or lack of anti-bullying policies [27].

Within India’s unique socio-cultural factors deeply entrenched gender norms, caste dynamics, and disparities in digital access—these elements may play a critical role within the ecological framework and its relevance and explanatory power. A culturally grounded application of the ecological model is therefore essential for designing effective, context-specific interventions. Therefore, research examining the role of contextual factors specific to India is important.

With this background, this study addresses the gaps by assessing the prevalence of physical and cyber bullying among school-going adolescents aged 13–17 in Himachal Pradesh, India, and to identifying risky and protective factors using an ecological framework [21]. Insights from this research could enhance the ongoing School Health and Wellness Program under *Ayushman Bharat* initiative by integrating anti-bullying measures within the existing relevant modules. This would supplement the Government of India’s ongoing efforts to address bullying and cyberbullying in schools, which include the mandatory anti-ragging committees and cyber-safety awareness campaigns in the selected states.

## Materials and methods

### Study design and setting

A cross-sectional design was used to conduct the self-administered school-based adolescent health survey among adolescent boys and girls (13–17 years) attending government schools in rural and urban areas across all 12 districts of Himachal Pradesh, India. Himachal Pradesh was selected because it has enabling education environment (928 government high schools and 1,869 government senior secondary schools); high enrolment and retention (nearly universal enrolment in primary education, and high retention up to higher grades) and Gender parity (Gender Parity Index, a ratio to measure gender equality in accessing education, of 1.01 at the secondary level and 1.03 at the higher secondary level); and state government preparedness for survey to improve adolescent health and wellbeing.

### Sample size and participant recruitment

Sample size was determined using the Cochran’s Sampling for infinite population [28]. To account for non-responses, incomplete responses, and consent refusals, an additional 15% was added to the initial sample size. The total sample of 7,588 adolescents were drawn from all 12 districts using the probability- proportional-to-size, taking into account school location (rural and urban), gender (boy and girl), school type (co-ed, exclusive boy’s or girl’s schools) and adolescents age (13–15 years and 16–17 years). To avoid over-representation, only one school from each school cluster (an administrative system for Indian schools) was selected. This approach facilitated a harmonised distribution of respondents across clusters, reduced clustering bias, and improved the overall representativeness of the sample.

**Fig 1:**
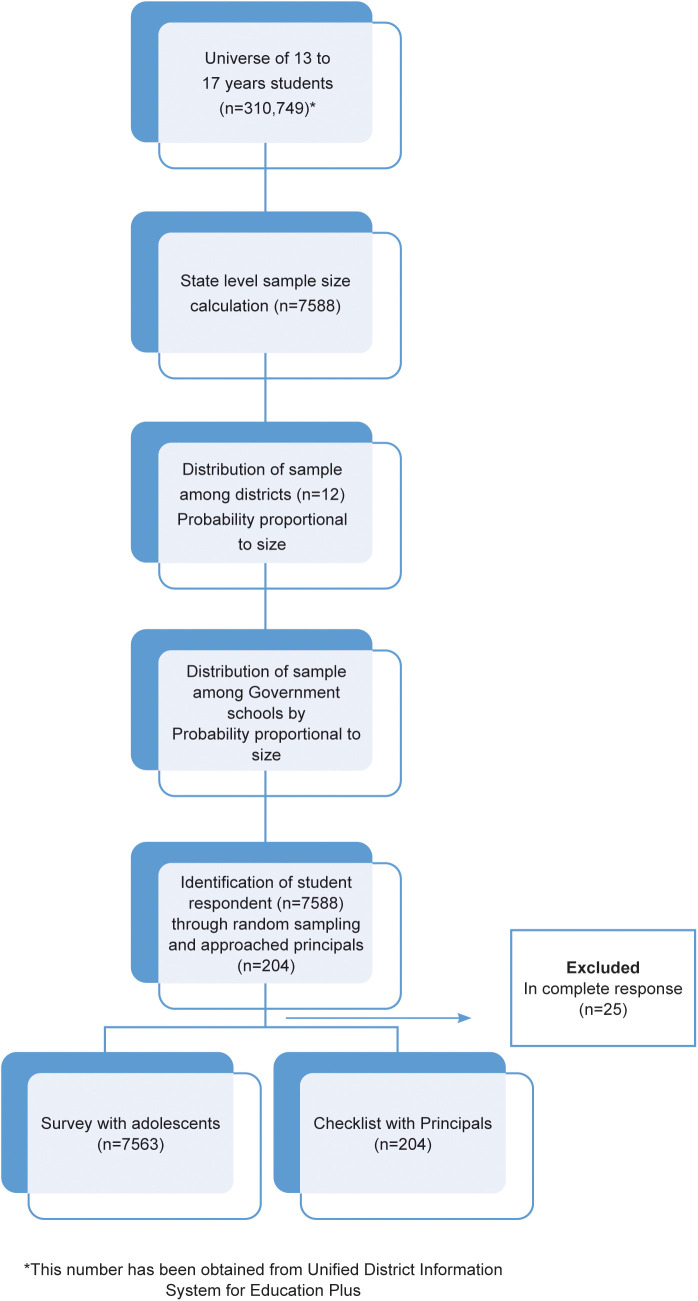
Schematic representation of sample selection.

Adolescents (n = 7588) were enrolled from 204 Government schools and the principals (n = 204) of these schools also participated in the survey (Fig 1). Fig 1 represents the schematic representation of the sample selection for the study. As per the PISA (Programme for International Student Assessment) taxonomy, Indian government schools correspond to PISA's category of public schools.

Adolescent provided personal insights of their health behaviors while the school principals gave perspective on the broader school environment and policies which influences student well-being. This approach complemented and strengthened data finding triangulation and validation.

### Data collection

Self-administered tools were developed for students and principals. The student survey was based on a framework of domains and questions adapted from the ‘Global School-based Student Health Survey [29]. The questionnaire covered a wide range of topics including dietary behavior, physical activity, hygiene, digital device use, connection with parents/guardians, health-seeking behavior, health screening, violence, substance use, reproductive and sexual health, menstrual hygiene, and mental health. Each survey question was mapped to the core domains and recommended indicators identified by Global Action for Measurement of Adolescent Health (GAMA) initiative [30]. This ensured standardized measurement across key areas such as mental health, substance use, violence etc.

For the school principals, ‘Global School Health Policies and Practices Survey’ tool [31] was contextualised to understand the implementation of school health policies aimed at promoting health and wellbeing of adolescents. Domains included school health coordination, healthy and safe school environment, violence and bullying, substance use prevention, implementation of government schemes, and physical education and activity. However, in this paper we present only the bullying domain.

Tools developed (English and Hindi) were pilot tested for content and face validity. Content validity was established by consulting thematic experts (n = 14) who provided feedback on the accuracy and relevance of the survey content, ensuring comprehensive coverage of all key areas. Face validity was assessed by involving boys and girls enrolled in class 7th to 12th (n = 90) from two districts representing both rural and urban setting. These students were asked to review the questionnaire items for clarity, relevance, and comprehensibility (S1 Text). This feedback was used to refine the wording and format of items to enhance the overall acceptability and interpretability of the survey for the sampled adolescent population. For group concurrent validity, face-to-face interviews were conducted with adolescents (n = 90). Feedback from both thematic experts and adolescent participants was incorporated into the final version of the tools ensuring the cultural and contextual appropriateness for the target population before full-scale data collection commenced. Both the tools were administered using KoBo collect [32], interface during November-December, 2023. Data were collected by research assistants who completed a 2-day training on data collection techniques and ethical considerations before commencing the survey. The survey was administered to the students with state and school approval, without disturbing the school schedule. Students took about 45–60 minutes and principal took 15 minutes to complete the survey. A total of 7563 adolescents and 204 school principals consented for participation.

### Variables

In this analysis, we examined bullying as the outcome variable, which includes both physical bullying (victim and perpetrator) and cyberbullying (victim and perpetrator). Potential factors associated with bullying were descriptively categorised within the ecological framework, variables were classified at the individual, family, peer and school system level [23,24,33–40]. The analysis was restricted to variables available in this survey and association established in literature. As the study did not collect peer-level data, this dimension could not be included in the analysis. For individual-level independent variables, age was dichotomized into 13–15 years and 16–17 years, while sex was categorized as male or female. Time spent on digital devices was classified as less than 8 hours or 8 hours or more per day. Variables such as consumption of junk food, skipping breakfast, owning a phone, substance use, history of sexual activity, smoking, feelings of depression, worry, nervousness or anxiety, difficulty in staying focused, involvement in physical fights, and feeling unsafe while going to school were dichotomized as yes or no.

At the family level, time spent with parents and parental supervision were categorized as Never/Rarely/Sometimes and Most of the time/Always. Parental occupation was classified into unskilled/semi-skilled, skilled/professional and self-employed. At the school level, the presence of school policies related to bullying was dichotomized as yes or no. Variables included in the study is provided in Table 1.

**Table 1:** Study Variables used in the analysis.

Variable	Survey Question	Response Option
**Outcome Variables**		
Cyber bullying perpetration	During the past 12 months, did you bully someone online?	Yes = 1, No = 0
Cyber bullying victimization	During the past 12 months, did someone bully you online?	Yes = 1, No = 0
Physical bullying perpetration	During the past 12 months, did you bully someone?	Yes = 1, No = 0
Physical bullying victimization	During the past 12 months, did someone bully you?	Yes = 1, No = 0
Explanatory Variables		
***Individual System***		
Gender	Sex of the respondent?	Boy = 1, Girl = 2 and Other = 3
Age	Age in completed years of the respondent	13-15 years = 1, 16-17 years = 2
Place of residence	Place of residence of the respondent?	Rural = 1, Urban = 2
Junk food consumption	1 Fried food During the past 7 days, how many times did you eat fried food such as *samosa*, *pakoda, Kachori, poori*, fried Chicken, fried fish, *mathri*, *bhuture* etc.? **2.** Soda beverage During the past 7 days, how many times did you take soda beverage (soft drinks) like Pepsi, Coca Cola, Mirinda, Sprite, 7UP, Limca etc.? **3.** High salt and fats During the past 7 days, how many times did you consume foods with high salt and fats such as chips/pizza/burger/ *bhujia/ namkeen* /*fan*/ *Chowmein* etc.?	No = 1 if did not consume or consumed 1–3 times fried food/ soda beverage/ high salt and fats during the past 7 days Yes = 2 if consumed 4–6 times fried food/ soda beverage/ high salt and fats during the past 7 days or 1 time per day or 2 times per day or, 3 times per day or 4 or more times per day If any of the items listed in column 2 (fried foods, soda, or high-salt and high-fat foods) were consumed as per the criteria for “Yes” the participant is classified as a junk food consumer.
Breakfast skipping	During the past 30 days, how often did you eat breakfast?	Yes = 1 if Never or Rarely or Sometimes No = 2 if Most of the times or Always
Hours of digital device usage	During the past 7 days, how many hours per day did you spent on digital device (example: television, mobile phone, table computer, laptop, tablet, etc.)?	<=8 hr per day = 1 if did not have any digital device or Less than 1 hour per day or 1 to 2 hours per day or 3 to 4 hours per day or 5 to 6 hours per day or 7 to 8 hours per day > 8ht per day = 2 if More than 8 hours per day
Feeling unsafe when going to school	During the past 30 days, on how many days did you not go to school because you felt you would be unsafe (passing comment, blocking the road, blocking the road by riding a bike/cycle in a dangerous manner, pulling chunari, pulling hair, touching the body, beating, abusing) at school or while going or coming back from school?	No = 1 if 0 days Yes = 2 if 1 day or 2 or 3 days or 4 or 5 days or 6 or more days
Substance use	Consumption of any of the following substances 1. smoke cigarette/ bidi/ hookah and/ or chewing tobacco in past 30 days 2. use e-cigarette in past 30 days 3. Consume alcohol in past 30 days 4. use ganja in past 12 months 5. use cocaine/ heroin (Chitta)/opium in past 12 months 6. use Hallucinogens (it is a swallowable pill, after taking which the person loses his balance) like LSD in past 30 days 7. Ever used a needle to inject any illegal drug into your body 8. use inhalants (solvent, gas from lighters, paint thinner in past 12 months 9. consume cough syrup without a prescription in past 12 months 10. sleeping pills like Panadol night, diazepam without a prescription	No = 1 if 0 times Yes = 1 if 1 or 2 times or 3–5 times or 6–9 times or 10–19 times or 20 or more times Substance use defined as consumption of the any of the substances (1–10)
Indulgence in physical fights	During the past 12 months, did you get into a physical fight?	Yes = 1, No = 0
Sexual intercourse	Have you ever had sexual intercourse?	Yes = 1, No = 0
Disappointed/ depressed/ hopeless	During the past 12 months, how often did you feel disappointed, depressed, hopeless or have little interest in or get little pleasure from doing things?	No = 1 if Never or Rarely or Sometimes Yes = 2 if Most of the times or Always
Worried	During the past 12 months, how often were you so worried about something that you either could not eat/ did not feel hungry, or ate too much?	No = 1 if Never or Rarely or Sometimes Yes = 2 if Most of the times or Always
Nervous or Anxious	During the past 12 months, how often did you feel nervous or anxious or not able to stop or control worrying?	No = 1 if Never or Rarely or Sometimes Yes = 2 if Most of the times or Always
Difficulty in focussing on homework (Possibly stressed)	During the past 12 months, how often did you have a difficult time staying focused on your homework or other tasks you had to do?	No = 1 if Never or Rarely or Sometimes Yes = 2 if Most of the times or Always
***Family System***		
Breadwinner occupation	Occupation of the main breadwinner of your family?	Unskilled/Semi-skilled = 1, Skilled/ Professional = 2, Self-employed = 3
Parent connectedness	During the past 30 days, how often did your parents and/or guardians spend time with you?	No = 1 if Never or Rarely or Sometimes Yes = 2 if Most of the times or Always
Parental supervision	During the past 30 days, how often did your parents and/or guardians try to know what you were doing in your free time?	No = 1 if Never or Rarely or Sometimes Yes = 2 if Most of the times or Always
***School System***		
Written policy/guideline/rule prohibiting bullying among students at school	Does your school have or follow a written policy/guideline/rule prohibiting bullying among students at school?	Yes = 1, No = 0

### Statistical analysis

Data was analyzed with STATA 18.5 version. The distribution of background characteristics has been represented in frequency and percentage. Bivariate analysis using chi-square test was performed to assess the association between various independent variables and bullying behaviours (physical and cyber) among adolescents, including both perpetrators and victims.

Given the hierarchical structure of the data, with students nested within schools, we initially fitted multilevel logistic regression models with a random intercept at the school level to account for clustering for each of the outcome variable separately. The null model indicated that school-level variation accounted for 7–10% of the total variance (ICC = 0.07–0.10) across bullying outcomes. As a robustness check, standard multivariate logistic regression models adjusted for the same covariates were also fitted. The adjusted odds ratios (aORs) and 95% confidence intervals (CIs) from both approaches were nearly identical, indicating minimal impact of clustering on the observed association. Therefore, for parsimony and clarity of presentation, results from the standard multivariate logistic regression are presented in the main text, with multilevel model estimates provided in the S2 Text.

Only variables showing significant associations in bivariate analyses (p < 0.05) were included in the multivariate logistic regression model [40–43]. We did not control for additional variables outside this selection. This approach reduced model complexity and avoided overfitting, ensuring that only the most relevant factors—those showing initial associations with the outcomes—were examined in the adjusted analysis.

Further, we assessed multi-collinearity among independent variables using variance inflation factor (VIF). The mean VIF ranged from 1.09 to 1.13, indicating no evidence of sticky collinearity among the independent variables. Additionally, Model fit was assessed using the likelihood ratio chi-square test and Pseudo-R². The likelihood ratio test with p value <0.05 indicated that the model with predictors fit the data significantly better than an empty model (model with no predictors). The proportion of variance in bullying experiences that could be explained by the predictors was reported (Table 4).

### Ethical consideration

The Institutional Review Board of Mamta Health Institute for Mother and Child, New Delhi approved the study (MIRB/September – 2023/009). Prior to data collection, written consent was obtained from school authorities. Passive consent [44] was obtained from parents or legal guardians for their child's participation in this survey. One week prior to the survey, a printed notification letter from the school principal was sent home to all eligible students. The letter detailed the overview of the study, its objectives, data collection timeline, assurance of confidentiality, and a statement emphasizing that participation is voluntary. It also included contact information for the research team and a section for passive parental consent, allowing parents or guardians to opt out by signing and returning a refusal form. In cases where parents were unable to read the letter, students were instructed to read it aloud to ensure the information was clearly conveyed and understood. Passive parental consent was deemed appropriate given the minimal risk nature of the study, the large school-based sample, and the need to ensure representative participation while minimizing administrative burden. The opt-out mechanism consisted of an information sheet accompanied by a brief opt-out form, which parents could sign and return to the school to indicate that they did not wish their child to participate.

Electronic assent was obtained from each student in the opening of the self-administered survey interface before data collection began.

## Results

### Demographic characteristics of the schools

A total of 204 government schools across all 12 districts of Himachal Pradesh were included in the study. Majority schools were coeducational institutions (85.8%), with a smaller proportion being boys-only (6.4%). Most schools (89.2%) were higher secondary schools. Majority (86.8%) were rural areas, aligned with state’s predominantly rural educational landscape (Table 2).

**Table 2:** Characteristics of Schools surveyed.

Variable	Frequency	Percentage
**School Type**		
Only boy’s school	13	6.4
Only girl’s school	16	7.8
Coeducational school	175	85.8
**Grades taught in school**		
Upper Primary School	4	2.0
High School	18	8.8
**Higher Secondary School**	182	89.2
School Location		
Rural	177	86.8
Urban	27	13.2

### Socio-demographic characteristics of the study participants

A total of 7,563 school-going adolescents participated in the survey, achieving a response rate of 99.96%, those students (n = 2) provided incomplete responses were removed from analysis. The gender distribution with 50.68% boys and 49.32% girls was nearly equal. The majority of respondents (67.08%) were aged 13–15 years and a significant proportion were residing in rural areas (88.42%), reflecting the demographic composition of Himachal Pradesh. The breadwinner for the student’s family worked in unskilled or semi-skilled occupations (49.53%), which may serve as a proxy for lower socioeconomic status (Table 3).

**Table 3:** Description of the study participation.

Variable	Frequency	Percentage
**Gender**		
Boys	3833	50.68
Girls	3730	49.32
**Age**		
13-15 years	5073	67.08
16-17 years	2490	32.92
**Residence**		
Rural	6687	88.42
Urban	876	11.58
**Family size** ^*^		
<=4	2536	33.53
>4	5027	66.47
**Parental occupation**		
unskilled/semi-skilled	3746	49.53
skilled/professional	2061	27.25
self-employed	1756	23.22

*Family size refers to the total number of individuals living in the household, including the adolescent respondent.

### Prevalence of bullying perpetrator and bullying victimization

Among 7563 adolescent respondents, 1392(18.41%) reported engaging in both form of bullying (physical or cyber), with 1056 (13.96%) engaged in physical bullying and 729 (9.64%) in cyberbullying. Among 1392 adolescents who reported engaging in either form of bullying, 393 (28.23%) were identified as perpetrators of both physical and cyberbullying. In contrast, 1180 adolescents (15.60%) reported being victims of either form of bullying, with 862 (11.40%) experiencing physical bullying and 596 (7.88%) reporting cyberbullying. Among these, 278 adolescents (23.56%) had been subjected to both forms of bullying (physical and cyberbullying) in the past 12 months (Table 4). The prevalence of bullying was significantly higher among boys, older adolescents, those living in urban areas, and individuals who consumed junk food or skipped breakfast. It was also more common among adolescents who spent significant time on digital devices, used substances, or history of sexual activity. Additionally, higher incidence of bullying was reported among those experiencing feelings of depression, anxiety, nervousness, or difficulty concentrating, as well as among adolescents who occasionally spent time with their parents or received limited parental supervision (Table 4).

**Table 4:** Prevalence of perpetrator and victims (both Physical and Cyber bullying).

Characteristics		Bullying				Bullying victimization				Total
		*Physical*		*Cyber*		*Physical*		*Cyber*		
		Yes (%)	*p-value*	Yes (%)	*p-value*	Yes (%)	*p-value*	Yes (%)	*p-value*	
Overall		1056 (13.96)	*NA*	729 (9.64)	*NA*	862 (11.40)	*NA*	596 (7.88)	*NA*	
Either physical or cyber		1392 (18.41)				*1180 (15.60)*				
Both physical and cyber		393 (28.23)				*278 (23.56)*				
**Individual level**										
Gender	Girl	375 (10.05)	<0.001	251 (6.73)	<0.001	274 (7.35)	<0.001	215 (5.76)	<0.001	3730 (49.32)
	Boy	681 (17.77)		478 (12.47)		588 (15.34)		381 (9.94)		3833 (50.68)
Age	13-15 years	692 (13.64)	0.25	439 (8.65)	<0.001	578 (11.39)	0.99	365 (7.19)	<0.01	5073 (67.08)
	16-17 years	364 (14.62)		290 (11.65)		284 (11.41)		231 (9.28)		2490 (32.92)
Residence	Rural	899 (13.44)	<0.001	620 (9.27)	<0.01	722 (10.8)	<0.001	500 (7.48)	<0.001	6687 (88.42)
	Urban	157 (17.92)		109 (12.44)		140 (15.98)		96 (10.96)		876 (11.58)
Consumption of Junk food	No	285 (10.41)	<0.001	174 (6.36)	<0.001	250 (9.13)	<0.001	161 (5.88)	<0.001	2738 (36.2)
	Yes	771 (15.98)		555 (11.5)		612 (12.68)		435 (9.02)		4825 (63.8)
Skipped breakfast	No	719 (12.38)	<0.001	489 (8.42)	<0.001	588 (10.12)	<0.001	389 (6.7)	<0.001	5809 (76.81)
	Yes	337 (19.21)		240 (13.68)		274 (15.62)		207 (11.8)		1754 (23.19)
Time spent on digital devise	<8 hr	1000 (13.49)	<0.001	679 (9.16)	<0.001	834 (11.25)	<0.01	567 (7.65)	<0.001	7413 (98.02)
	>=8 hr	56 (37.33)		50 (33.33)		28 (18.67)		29 (19.33)		150 (1.98)
Feeling unsafe while going to school	No	NA	NA	NA	NA	491 (8.39)	<0.001	337 (5.76)	<0.001	5853 (77.39)
	Yes	NA		NA		371 (21.7)		259 (15.15)		1710 (22.61)
Owning a phone	Yes	408 (20.17)	<0.001	372 (6.71)	<0.001	539 (9.73)	<0.001	319 (5.76)	<0.001	2023 (26.75)
	No	648 (11.7)		357 (17.65)		323 (15.97)		277 (13.69)		5540 (73.25)
Substance use	No	545 (10.38)	<0.001	384 (7.31)	<0.001	460 (8.76)	<0.001	321 (6.11)	<0.001	5251 (69.43)
	Yes	511 (22.1)		345 (14.92)		402 (17.39)		275 (11.89)		2312 (30.57)
Ever had sexual intercourse	No	893 (12.55)	<0.001	607 (8.53)	<0.001	731 (10.28)	<0.001	490 (6.89)	<0.001	7113 (94.05)
	Yes	163 (36.22)		122 (27.11)		131 (29.11)		106 (23.56)		450 (5.95)
Felt depressed	No	879 (12.77)	<0.001	602 (8.75)	<0.001	711 (10.33)	<0.001	480 (6.97)	<0.001	6883 (91.01)
	Yes	177 (26.03)		127 (18.68)		151 (22.21)		116 (17.06)		680 (8.99)
Felt worried	No	930 (13.14)	<0.001	627 (8.86)	<0.001	748 (10.57)	<0.001	510 (7.21)	<0.001	7078 (93.59)
	Yes	126 (25.98)		102 (21.03)		114 (23.51)		86 (17.73)		485 (6.41)
Felt nervous or anxious	No	918 (12.93)	<0.001	626 (8.82)	<0.001	745 (10.49)	<0.001	503 (7.08)	<0.001	7100 (93.88)
	Yes	138 (29.81)		103 (22.25)		117 (25.27)		93 (20.09)		463 (6.12)
Difficulty in stay focused	No	836 (12.44)	<0.001	575 (8.55)	<0.001	667 (9.92)	<0.001	456 (6.78)	<0.001	6722 (88.88)
	Yes	220 (26.16)		154 (18.31)		195 (23.19)		140 (16.65)		841 (11.12)
Indulged in Physical fight	No	459 (7.9)	<0.001	397 (6.83)	<0.001	424 (7.29)	<0.001	316 (5.44)	<0.001	5813 (76.86)
	Yes	597 (34.11)		332 (18.97)		438 (25.03)		280 (16)		1750 (23.14)
**Family level**										
Time spent with parents	Never/Rarely/Sometimes	304 (20.69)	<0.001	217 (14.77)	<0.001	261 (17.77)	<0.001	188 (12.8)	<0.001	1469 (19.42)
	Most of the time/always	752 (12.34)		512 (8.4)		601 (9.86)		408 (6.7)		6094 (80.58)
Patental supervision	Never/Rarely/Sometimes	526 (16.41)	<0.001	347 (10.83)	<0.01	417 (13.01)	<0.001	291 (9.08)	<0.001	3205 (42.38)
	Most of the time/always	530 (12.16)		382 (8.77)		445 (10.21)		305 (7)		4358 (57.62)
Parental occupation	skilled/professional	296 (14.36)	0.75	179 (8.69)	0.03	428 (11.43)	0.43	297 (7.93)	0.9	2061 (27.25)
	unskilled/semi-skilled	512 (13.67)		396 (10.57)		247 (11.98)		165 (8.01)		3746 (49.53)
	self-employed	248 (14.12)		154 (8.77)		187 (10.65)		134 (7.63)		1756 (23.22)
**School level**										
written policy/guideline/rule prohibiting bullying	No	34 (11.97)	0.32	32 (11.27)	0.34	33 (11.62)	0.9	20 (7.04)	0.59	284 (3.76)
	Yes	1022 (14.04)		697 (9.58)		829 (11.39)		576 (7.91)		7279 (96.24)

### Determinants for indulging in bullying with others (Physical and Cyber bullying)

**Fig 2:**
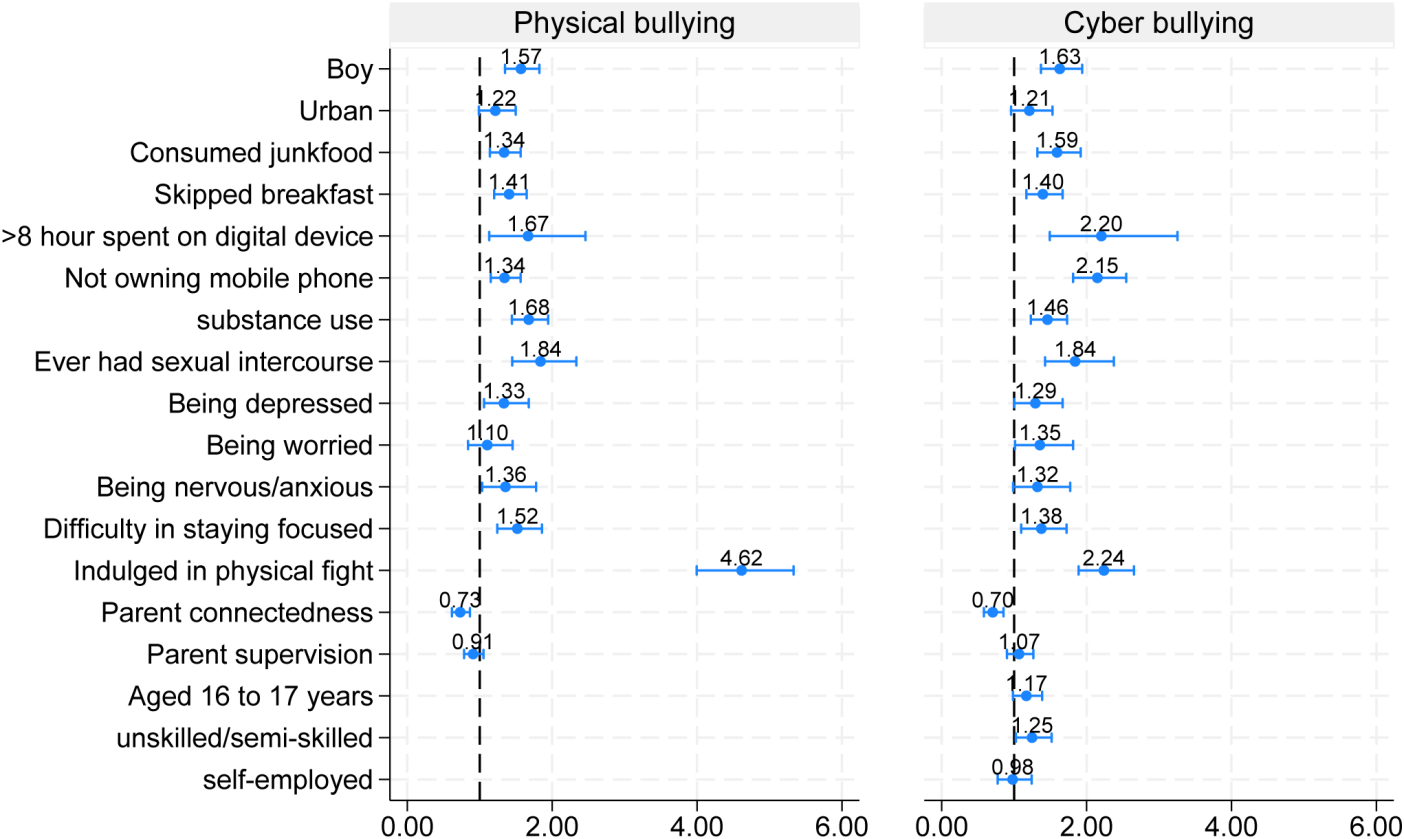
Predictors of physical and cyber bullying perpetrator (adjusted OR from Multivariable logistic regression model).

*Individual Level:* Boys had higher odds for perpetrating both physical bullying (aOR = 1.57; 95% CI: 1.35–1.82; p < 0.01) and cyberbullying (aOR = 1.63; 95% CI: 1.37–1.94; p < 0.01) compared to girls. Adolescents who consumed junk food in the past week were 1.3 to 1.6 times more likely to engage in bullying others (both physical and cyber). Those who skipped breakfast had a 1.4 times higher likelihood of bullying physically. Adolescents who spent 8 or more hours daily on digital devices were more likely to bully others physically (aOR = 1.67; 95% CI: 1.13–2.46; p < 0.01) and through cyber means (aOR = 2.2; 95% CI: 1.49–3.25; p < 0.01). Adolescents who used substances were significantly more likely to be engaged in both forms of bullying (aOR= 1.68; 95% CI:1.45, 1.95; p value <0.01 for physical bullying) and (aOR= 1.46; 95% CI: 1.23, 1.73; p value <0.01 for cyberbullying). Furthermore, those who had history of sexual activity were 84% more likely to bully others either physical (AOR = 1.84; 95% CI: 1.45,2.33; p value <0.01) or through cyber means (aOR=1.84; 95% CI: 1.43,2.38; p value <0.01). Adolescents who experienced hopelessness/ disappointed in last 12 months were more likely to bully others physically (aOR = 1.33; 95% CI 1.06, 1.68; p value<0.05) and cyber space (aOR = 1.29; 95% CI 1.00, 1.67; p value0.048). Similarly, adolescents who reported experiencing difficulty in staying focused on homework were more likely to bully others physical and cyber means (aOR = 1.52; 95% CI 1.24, 1.86; p value<0.01) and (aOR = 1.38; 95% CI 1.10, 1.72; p value<0.01), respectively. Additionally, those who reported with nervousness/ anxiety had higher odds of physically bullying others (aOR = 1.36; 95% CI 1.03, 1.78; p < 0.05), while adolescents who were worried were more likely to cyberbully (aOR = 1.35; 95% CI 1.01, 1.81; p < 0.05). Adolescents who reported being engaged in physical fights were 4.62 times more likely to physically bully others and 2.24 times more likely to engage in cyberbullying, compared to those who did not participate in physical fights. However, adolescents who did not own a personal phone was also associated with bullying, with odds of 1.3 times (95% CI: 1.15–1.57) for physical bullying and 2.15 times (95% CI: 1.81–2.55) for cyberbullying (Table 5; Fig 2).

**Table 5:** Factors (risky and protective) of perpetrator and victims (both Physical and Cyber bullying).

Characteristics		Bullying		Bullying victimization	
		*Physical*	*Cyber*	*Physical*	*Cyber*
		aOR [95% CI]	aOR [95% CI]	aOR [95% CI]	aOR [95% CI]
**Individual level**					
Gender	Girl	Ref	Ref	Ref	Ref
	Boy	1.57** [1.35,1.82]	1.63** [1.37,1.94]	1.91** [1.62,2.25]	1.41** [1.17,1.71]
Age	13-15 years	–	Ref		Ref
	16-17 years	–	1.17 [0.98,1.39]		1.12 [0.93,1.35]
Residence	Rural	Ref	Ref	Ref	Ref
	Urban	1.22 [0.99,1.50]	1.21 [0.96,1.53]	1.35** [1.09,1.67]	1.27 [0.99,1.63]
Consumption of Junk food	No	Ref	Ref	Ref	Ref
	Yes	1.34** [1.14,1.56]	1.59** [1.32,1.92]	1.16 [0.98,1.37]	1.25* [1.02,1.52]
Skipped breakfast	No	Ref	Ref	Ref	Ref
	Yes	1.41** [1.20,1.65]	1.4** [1.17,1.67]	1.33** [1.12,1.58]	1.47** [1.21,1.78]
Time spent on digital devise	<8 hr	Ref	Ref	Ref	Ref
	>=8 hr	1.67** [1.13,2.46]	2.2** [1.49,3.25]	0.69 [0.43,1.11]	1.11 [0.70,1.77]
Feeling unsafe while going to school	No	NA	NA	Ref	Ref
	Yes	NA	NA	1.93** [1.64,2.27]	1.83** [1.51,2.21]
Owning a phone	Yes	Ref	Ref	Ref	Ref
	No	1.34** [1.15,1.57]	2.15** [1.81,2.55]	1.22* [1.03,1.44]	1.87** [1.55,2.25]
Substance use	No	Ref	Ref	Ref	Ref
	Yes	1.68** [1.45,1.95]	1.46** [1.23,1.73]	1.38** [1.17,1.63]	1.2 [0.99,1.45]
History of sexual activity	No	Ref	Ref	Ref	Ref
	Yes	1.84** [1.45,2.33]	1.84** [1.43,2.38]	1.53** [1.19,1.96]	1.85** [1.41,2.42]
Felt depressed	No	Ref	Ref	Ref	Ref
	Yes	1.33* [1.06,1.68]	1.29 [1.00,1.67]	1.42** [1.12,1.80]	1.48** [1.14,1.93]
Felt worried	No	Ref	Ref	Ref	Ref
	Yes	1.1 [0.84,1.45]	1.35* [1.01,1.81]	1.3 [0.98,1.72]	1.2 [0.88,1.64]
Felt nervous or anxious	No	Ref	Ref	Ref	Ref
	Yes	1.36* [1.03,1.78]	1.32 [0.99,1.77]	1.25 [0.94,1.66]	1.34 [0.99,1.82]
Difficulty in stay focused	No	Ref	Ref	Ref	Ref
	Yes	1.52** [1.24,1.86]	1.38** [1.10,1.72]	1.61** [1.31,1.98]	1.52* [1.20,1.92]
Indulged in Physical fight	No	Ref	Ref	Ref	Ref
	Yes	4.62** [4.00,5.33]	2.24** [1.89,2.65]	3.04** [2.60,3.56]	2.26** [1.88,2.71]
**Family level**					
Time spent with parents	Never/Rarely/Sometimes	Ref	Ref	Ref	Ref
	Most of the time/always	0.73** [0.62,0.87]	0.7** [0.58,0.85]	0.65** [0.55,0.78]	0.67** [0.55,0.82]
Parental supervision	Never/Rarely/Sometimes	Ref	Ref	Ref	Ref
	Most of the time/always	0.91 [0.78,1.05]	1.07 [0.90,1.27]	1.02 [0.87,1.19]	1.02 [0.85,1.22]
Parental occupation	skilled/professional	–	Ref	–	–
	unskilled/semi-skilled	–	1.25* [1.02,1.52]	–	–
	self-employed	–	0.98 [0.77,1.24]	–	–
**School level**					
written policy/guideline/rule prohibiting bullying	No	–	–	–	–
	Yes	–	–	–	–
N		7563	7563	7563	7563
LR chi2 (*P value*)		1007.82(<0.001)	618.7(<0.001)	756.54(<0.001	526.71(<0.001)
Pseudo R2		0.17	0.13	0.14	0.13
Mean VIF		1.09	1.13	1.1	1.1
** p < .01, * p < .05					

*Family Level***:** whose parents were engaged in unskilled occupations were significantly associated with cyberbullying perpetration (aOR = 1.25; 95% CI: 1.02–1.52) compared to those whose parents were in professional occupations. Conversely, parental connectedness was found to be protective, adolescents whose parents spent time with them showed negative association with bullying others physically (aOR = 0.73; 95% CI: 0.62–0.87; p < 0.01) and cyberbullying (aOR = 0.70; 95% CI: 0.58–0.85; p < 0.01) (Table 5; Fig 2).

*School Level:* Presence of guidelines against bullying in school was insignificant in bivariate analysis hence was not included in the multivariable analysis (Table 5).

### Factor associated with bullying victimization

**Fig 3:**
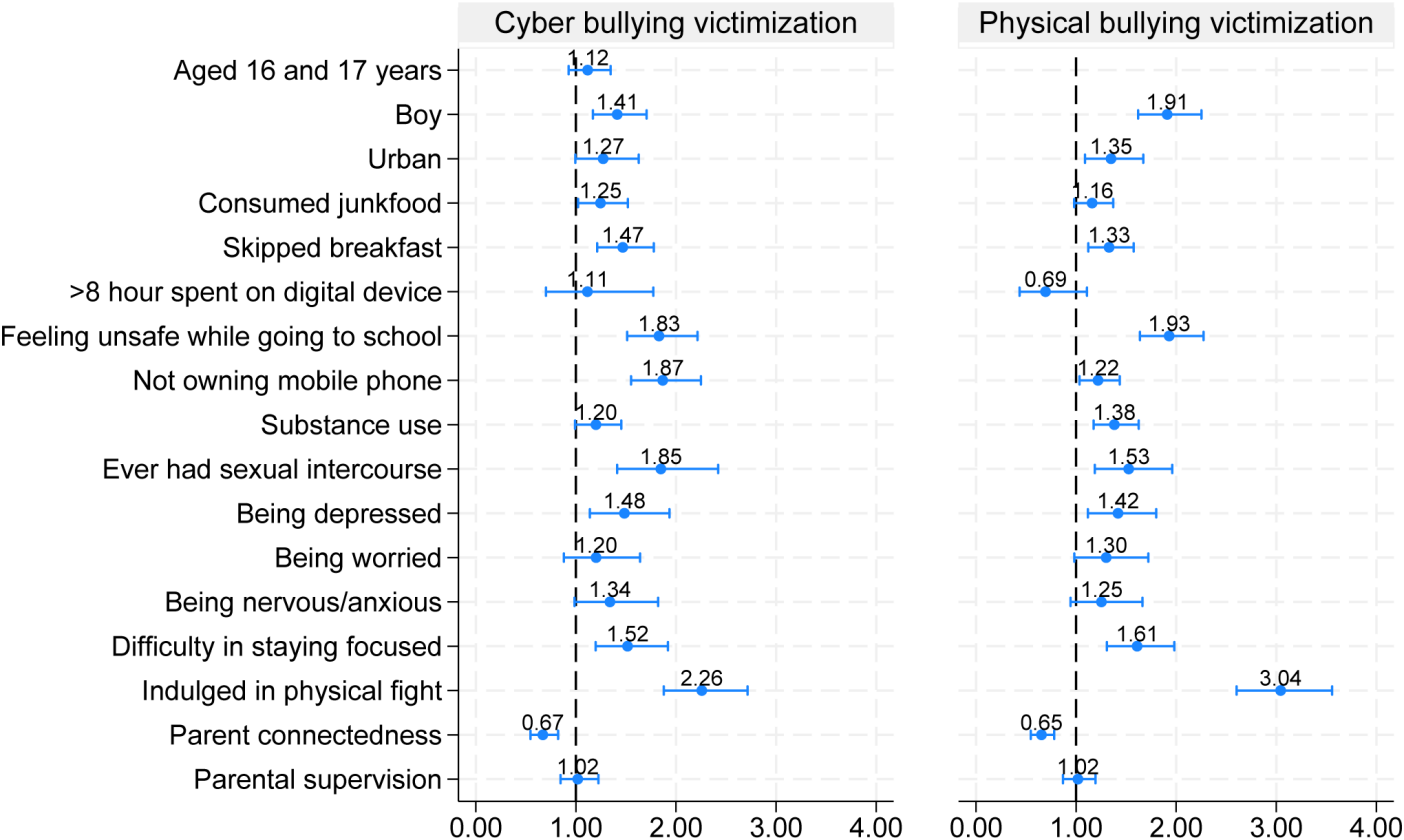
Predictors of physical and cyber bullying victimization (adjusted OR from Multivariable logistic regression model).

*Individual Level:* Boys were significantly higher odds of being bullied both physical (aOR=1.91; 95% CI: 1.62, 2.25; p < 0.01) and cyber (aOR=1.41; 95% CI: 1.17, 1.71; p < 0.01) compared to girls. Urban adolescents compared to rural faced a higher odds of being bullied physically (aOR=1.35; 95% CI: 1.09, 1.67; p < 0.01), however no significant association was observed for cyberbullying. Dietary habits emerged as a significant risk factor for being bullied. Adolescents who consumed junk food more than 4–6 times a week were 25% more likely to experience cyberbullying (aOR=1.25; 95% CI: 1.02, 1.52; p < 0.05). Similarly, adolescents who skipped breakfast were 33% more likely to be physically bullied and 47% more likely to be cyberbullied. (Table 5; Fig 3).

Adolescents who felt unsafe on their way to school were associated significantly with experiencing physical (aOR=1.93; 95% CI: 1.64, 2.27; p < 0.01) and cyberbullying (aOR=1.83; 95% CI: 1.51, 2.21; p < 0.01). Use of any substance increased odds of being physically bullied by 38% but had no significant effect on cyber bullying. Adolescents who had history of sexual activity had an increased odds of being victims of both physical (aOR=1.53; 95% CI: 1.19, 1.96; p < 0.01) and cyberbullying (aOR=1.85; 95% CI: 1.41, 2.42; p < 0.01). Similarly, those who reported feeling hopelessness/disappointment, and struggled with focus (possibly stressed), or involving in physical fights in the past 12 months was significantly associated with higher likelihood of being victims of both physical and cyberbullying (Table 5; Fig 3).

*Family Level:* Parental connectedness was found to be a protective factor against bullying victimization. Adolescents who reported that their parents spent time with them had significantly lower odds of experiencing physical bullying (aOR=0.65; 95% CI: 0.55, 0.78; p < 0.01) and cyberbullying (aOR=0.67; 95% CI: 0.55, 0.82; p < 0.01) (Table 5) (Fig 3).

**Fig 4:**
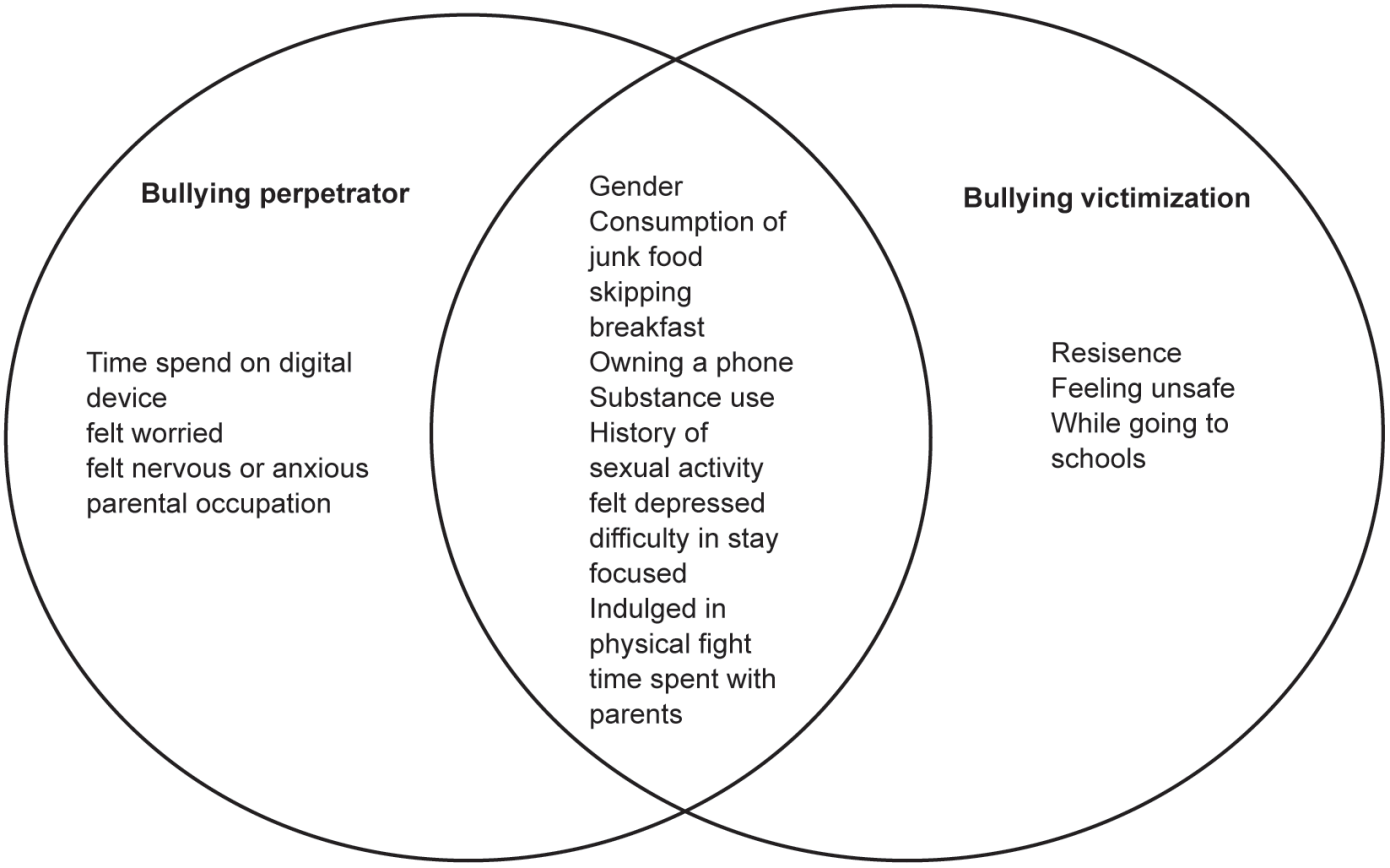
Overlapping and Distinct Factors Significantly Associated with Bullying victimization and Bullying perpetrator.

The Venn diagram (Fig 4) summarises illustrates the distinct and overlapping association of bullying perpetration and victimization among adolescents. Unique to perpetrators were factors like time spent on digital devices, feeling worried or anxious, and parental occupation, while urban residence and feeling unsafe on the way to school were specific for victims. Factors—including gender, substance use, mental health indicators, risky behaviors, and family connectedness—were common to both groups. These findings highlight the multifaceted nature of bullying involvement and the importance of addressing shared and role-specific risk.

## Discussion

This study is unique in its focus on early adolescents (13–17 years) experiencing bullying across multiple contexts, in both physical and online environments. To the best of our knowledge, it is the first study from India to comprehensively assess both bullying victimization and perpetration at the state level, using an ecological framework descriptively to explore associated factors.

We found that that overall, 18.41% adolescents reported having bullied someone (physical: 14% and online: 9.64%). Similarly, the bullying victimization in our study was 15.6% (Physical: 11.40%, cyberbullying: 7.88%). These findings indicate that both perpetration and victimization are prevalent among school-going adolescents in Himachal Pradesh, aligning with evidence observed across various parts of India. A systematic review [4] reported that bullying perpetration rates ranged from 7% [45] to 31% [46], and bullying victimization ranged from 9% [46] to 80% The wide variation highlights the differences in definitions, sampling techniques, and cultural factors across studies. Our results falling in the lower to mid-range, indicate a moderate level of bullying experiences among the surveyed adolescents. This could be due to underreporting, limited understanding of what constitutes bullying, or the acceptance of such behavior as normal. Importantly, findings show that while cyberbullying is somewhat less common than physical bullying, it remains significant, reflecting the growing digital medium involvement of adolescent bullying.

Study findings highlight the need for gender-sensitive bullying prevention, as boys show stronger links to both perpetration and victimization. This pattern is consistent with prior research indicating that boys are more likely to engage in direct, physical, and overt forms of bullying [4]. These gendered patterns suggest the distinct mechanisms through which bullying manifests among boys and girls needs to be focussed.

The ecological framework allowed us to descriptively examine how various factors positioned at the individual, family, and school level for bullying behaviors. These findings suggest that bullying has a broader social ecology including family dynamics, and school policies rather than being solely the result of individual factors.

### Individual level

The factors identified in our study are consistent with findings from other regions, including Ontario, Malawi, Nepal, and West Africa, where similar associations with bullying behaviors have been reported [36,47–50]. Notably unhealthy dietary habits such as frequent junk food consumption and skipping breakfast. These findings align with previous findings suggesting that poor nutrition may be associated with behavioral and emotional dysregulation, which in turn can contribute to increased aggression or vulnerability to peer victimization. For instance, breakfast skipping has been linked to lower energy levels, irritability, reduced cognitive performance, and poor mood regulation, all of which may impair social functioning and heighten conflictual peer interactions [51,52]. Furthermore, higher intake of junk food has been associated with poorer mental health outcomes, including symptoms of depression and anxiety [53], which may contribute to maladaptive peer interactions.

In addition, prolonged use of digital devices was associated with increased odds of bullying behaviour. A meta-analysis published in 2020 concluded that excessive screen time (≥2 hours/day) was associated with a 21% higher risk of bullying victimization. This may be because screen time exposes adolescents to violent or harmful online content exploration, reduces opportunities for positive social interactions, and impairs sleep quality, thereby contributing to irritability, impulsive behaviour, and decreased empathy—all of have been shown to be relevant to bullying behavior [54–57]. It also lead to greater exposure to risky online environments, where adolescents may either become targets of cyber aggression or engage in such behaviors themselves [54,58].

Furthermore, our study identified several mental health concern such as feeling of hopelessness and anxiety/ nervousness as significantly associated with increased likelihood of bullying involvement, either as a perpetrator or victim. Emotional distress, such as hopelessness and anxiety, may impair adolescents’ coping mechanisms, making them more vulnerable to peer aggression or more likely to externalize their distress through bullying others [59]. In some cases, adolescents may adopt bullying as a maladaptive realignment to regain control, assert dominance, or displace emotional turmoil, thereby perpetuating a cycle of aggression and victimization [25]. Furthermore, internalizing symptoms like anxiety or depression have shown to mediate the association between exposure to bullying and poor psychosocial outcomes [60].

Prevailing risk-taking behaviors including substance use, history of being sexually activity early, and involvement in physical fights were significantly associated with bullying perpetration. These behaviors often emerge from broader patterns of impulsivity, poor emotion regulation, and externalizing tendencies, all are linked to bullying behavior. substance use, for instance, may impair judgment and reduce inhibition, leading to greater likelihood of engaging in or tolerating aggression [61].

Given the links between bullying involvement and key individual-level factors, policy action must prioritize the integration of comprehensive health and psychosocial support services within school systems. Evidence shows that well-designed, school-based anti-bullying interventions are effective in significantly reducing both perpetration and victimization [62,63]. *Ayushman Bharat* School Health and Wellness Programme [64] currently addresses nutrition, mental well-being, substance misuse, sexual and reproductive health. This programme can be an effective platform to deliver targeted interventions. Therefore, its school curriculum should be updated to incorporate comprehensive content on bullying, including cyberbullying, under the component “Promotion of Safe Use of Internet, Gadgets, and Media”. Sensitising adolescents to the serious consequences of cyberbullying, while equipping them with available resources such as the ‘National Cyber Crime Reporting Portal [65] would empower students to recognise and respond appropriately to online harassment, contributing to a safer and more supportive environment. Additionally activities need to be included in the curriculum to equip students with the skills (social, crisis management and conflict resolution skills) [66]. Similar efforts have been taken by countries like Ireland, Philippines [67].

In light of our study findings, there is a pressing need to expand and enrich the programe to explicitly include bullying prevention and intervention, given its strong association with these domains. For instance, nutrition modules (Module 6, “Nutrition, Health, and Sanitation,” and Module 8, “Promotion of Healthy Lifestyle”) could be strengthened by adding content on the importance of breakfast. Similalry to discourage screen behaviour, it is essential to incorporate physical activity across all themes. Additionally, within substance misuse module, there is a need to add information on available services like National Tobacco Quit Line (1800 112 356) [68], mCessation (011–22901701) [69], linkages with nearest Adolescent Friendly Health Clinic, Health and Wellness Centers, and existing de-addiction centers [70] which can further aid in preventing substance misuse or supporting adolescents in quitting.

Bullying was more prevalent among students who had history of sexual activity, therefore it is crucial to include comprehensive sexuality education [71], to help adolescents understand sexuality issues and cope up with these rather than bullying others. Moreover, there is a critical need to add content on cyberbullying.

Many studies emphasize that owning a personal phone often increases risk of cyberbullying. An Indian study on school and college‑going adolescents reported that owning a smartphone was associated with increased risk of being cyberbullied [36]. In several European countries, use of mobile internet technologies and frequent online interaction via devices were linked to elevated rates of cyber victimization and perpetration [72]. However, our finding highlight that adolescents without a personal phone are more likely to bully or be bullied. This not only reflect shared family ownership but also the risks of digital exclusion. Phones serve as vital tools for peer communication and social inclusion [73], and lack of access can lead to social isolation and marginalization [74] that heightens the risk of bullying. This exclusion increases vulnerability to bullying, as these adolescents are often left out of digital support networks and have limited means to report or seek help [75]. Additionally, feelings of frustration and exclusion may prompt some to engage in bullying as a way to assert control [76]. Gender norms and constructs of masculinity further shape these dynamics, particularly in the Indian context, where boys are often socialized to be tough, self-reliant, and emotionally restrained, which may make them more reluctant to report victimization or seek help, thereby prolonging exposure to cyberbullying [77,78]. Reporting bias may also have also influenced the observed patterns, as boys tend to underreport emotional distress, while adolescents with limited digital access may either over- or under-perceive bullying experiences depending on their exposure and awareness [79,80]. Another possible explanation for this inconsistency is that owning a phone empowers adolescents with essentials skills like resilience and self-advocacy, that are essential in navigating social challenges like bullying [81]. However, this mechanism can only be confirmed by further exploration through scientific research.

### Family level

Parental connectedness in this study was a protective factor against bullying behaviour. Similar patterns were highlighted by others [82–85] who reported that stronger parental attachment correlates with lower bullying involvement. A meta-analysis [86] concludes that bullying prevention programs incorporating parental involvement are notably more successful in decreasing both bullying behavior and victimization. Thus Strengthening parent-child relationships should be an integral part of any anti-bullying intervention. *Ayushman Bharat* School Health and Wellness Program provides an opportunity to actively sensitised and guide parents, empowering them to play the proactive role in preventing bullying and promote positive behaviour among their children. Involving parents through information meetings, workshops, or communication sent home has been effective, as documented in a meta-analysis [87]. Similar approach can be adopted in reaching parents through *Ayushman Bharat* School Health and Wellness Programme.

### School level

While most schools in our study reported having anti-bullying policies, we found no significant association between the presence of such policies and reduced bullying behaviour pattern. This aligns with previous research suggesting that mere existence of a policy does not ensure effectiveness. Evidence indicates that policy quality—including clear definitions, reporting mechanisms, and consequences, significantly influences outcomes [88,89]. However, these policy-level variables were not assessed in depth in the present study, limiting our ability to evaluate school-level implementation and impact.

School policy should incorporate actionable steps inviting counsellors, medical officers, or community health officers, to engage with the school and community in anti-bullying efforts as part of the existing *Ayushman Bharat* School Health and Wellness Programme. These professionals can play a pivotal role in educating and sensitising parents, teachers, school management, and community members about the impact of bullying, its long-term consequences, and the importance of making safe, healthy choices [21]. This approach fosters collaboration among key stakeholders, reinforcing a shared commitment to promoting a safe and inclusive environment for all students.

In addition, it is crucial to regularly monitor bullying trends among adolescents within the national *Ayushman Bharat School Health and Wellness Programme*. This will help inform and guide the development and strengthening of state-level programmes and policies. Countries including Benin, Mauritius, Namibia, Seychelles, Argentina, Egypt and Jordan are already monitoring bullying at periodic intervals [51].

## Strength and limitations

The primary strengths of this study is that it provides estimation of the prevalence of self-reported bullying and its determinants among adolescents (13–17 years) from the state of Himachal Pradesh. During the critical developmental stage of adolescence, the instability of emotional development and the ongoing process of identity formation can make individuals particularly vulnerable to becoming either perpetrators or victims of bullying. The study utilized methodology of the Global School-based Health Survey, which employs a validated, collaborative, standardised questionnaire. We used a representative datasetwith a high survey response rate. Additionally, rigorous data collection methods were adopted with a reliable and standardized questionnaire, which ensured robust and consistent results.

While our study provides valuable insights, it also has some limitations. First, this analysis primarily on bullying and its proximate determinants, it did not account for the overall health and wellbeing. Secondly, due to the cross-sectional design, it is not possible to establish causal relationships between variables. Thirdly, as the data were self-reported, it may have introduced reporting and recall biases. However, since all the data collection process was anonymised and voluntary for the participants, this error would have had a minimal effect on the results. Additionally, the use of self-reported surveys may have led to underreporting of bullying experiences among girls or marginalized groups, potentially due to social stigma, fear of retaliation, or limited awareness and empowerment. Importantly, this analysis used data from the larger school-based adolescent health survey and lacked data on peer networks, peer attitudes, or peer behaviors within the context of bullying. This limitation significantly constrained the ecological model’s ability to holistically capture the social environment influencing the outcomes of interest. Adolescents are highly responsive to peer approval and social status, and when peers disapprove of bullying or actively intervene, the likelihood of bullying decreases [90]. Also, more variables for school level including school climate, peer norms, staff responsiveness, anti-bullying policy implementation could have added comprehensive understanding. Additionally, the generalizability of the study is limited, as it was conducted only in government and government aided schools of Himachal Pradesh.

## Conclusion

Study findings highlight the significant prevalence of bullying, both as victimization and perpetration, and underscore the importance of addressing the diverse micro, meso and macro factors driving these behaviors at the individual, family, and school level. Strengthening the national *Ayushman Bharat School Health and Wellness Programme* content by addressing specific issues such as bullying, substance use, unsafe sexual behavior, importance of regular nutritious meals and adding skill based activities could further protect adolescents. Future longitudinal research evaluating the effectiveness of such strengthened intervention in reducing bullying would be valuable.

## Supporting information

S1 Text Qualitative pilot-testing guide for a school-based adolescent health survey. (DOCX)

S2 Text Model Selection and Rationale. (DOCX)
