# Supplementary material for: Bullying among students ecological insights from a school based adolescent health survey, Himachal Pradesh, India
Source: PLoS One. 2026 Apr 2;21(4):e0345468. doi: 10.1371/journal.pone.0345468 (PMC13046284; doi:10.1371/journal.pone.0345468)
Supplement: S1 Text — (DOCX) [file pone.0345468.s001.docx]

**Qualitative Pilot-Testing Guide for School based Adolescent Health Survey**

**Aim of Pilot Testing:** To gather feedback from adolescents on how acceptable, understandable, and clear the survey questions are, before using it in the main study in Himachal Pradesh.

**Student ID (To be filled by field staff)**

**District Name:**

**Interviewer:**

**Start Time:**

**End Time:**

**Date:**

**Instructions**

### **General Instructions**

- **Purpose** – Explain that the pilot aims to test the clarity, relevance, and flow of the questionnaire—not to collect actual data for analysis.
- **Confidentiality** – Emphasize that responses are confidential and will not affect participants in any way.
- **Environment** – Conduct the pilot in a quiet, comfortable, and distraction-free setting similar to the planned main study environment.

### **During the Pilot**

1. **Observation** – Note participants’ non-verbal cues (confusion, hesitation, discomfort) as they respond.
2. **Comprehension Check** – Ask if any items were confusing, difficult, or repetitive.
3. **Cultural Relevance** – Check whether examples, terms, and response options are understandable and appropriate for the local adolescent context.
4. **Flow and Transitions** – Observe if the sequence of questions feels logical and engaging
5. Please note that the discussion of each section—including overall acceptability, wording and language, interpretability, and flow—should be conducted immediately after the completion of each module to minimize recall bias.

### **After Completion**

1. **Feedback Interview** – Conduct short interviews (using your guide) to gather opinions on:
   - Clarity of wording and language
   - Ease of understanding
   - Comfort level with sensitive questions
   - Relevance and completeness of response options
   - Suggestions for improvement
2. **Field Notes** – Document observations and participants’ comments systematically.

**Section 1: Overall Acceptability**

1. How did you feel about answering the survey?
   - (Prompt: Was it interesting, boring, too long, too personal, etc.?)
2. Were there any questions you felt uncomfortable or hesitant to answer?
   - (If yes, which ones and why?)
3. Did you feel that the topics covered were relevant and appropriate for students your age?
   - (Probe: Anything that didn’t feel suitable?)

#### ****Section 2: Wording and Language****

1. Were there any words, phrases, or sentences that were difficult to understand?
   - (Ask for examples and suggestions for simpler wording.)
2. Did any questions sound confusing or unclear in what they were asking?
   - (Probe for specific examples.)
3. Did the language used feel age-appropriate and relatable?
   - (Prompt: Did it sound too formal, too technical, or just right?)
4. Do you feel any questions or response options need further explanation or elaboration?
   - (Probe for specific examples.)

#### ****Section 3: Interpretability and Flow****

1. Were there any questions that you interpreted differently from what you think was intended?
   - (Probe: Can you explain how you understood that question?)
2. Did the order or flow of questions make sense to you?
   - (Prompt: Did it feel logical, or did it jump between unrelated topics?)
3. Were the response options (e.g., multiple-choice answers) easy to choose from and clear?
   - (Probe: Did any options feel missing or confusing?)

#### ****Section 4: Final Thoughts****

1. Overall, what changes would you suggest to make this survey easier or more comfortable for adolescents like you to complete?
2. Would you be willing to take part in a similar survey in the future? If yes, Why or If no, why not?
