# Supplementary material for: Bullying among students ecological insights from a school based adolescent health survey, Himachal Pradesh, India
Source: PLoS One. 2026 Apr 2;21(4):e0345468. doi: 10.1371/journal.pone.0345468 (PMC13046284; doi:10.1371/journal.pone.0345468)
Supplement: S2 Text — (DOCX) [file pone.0345468.s002.docx]

**Model Selection and Rationale**

Table1 shows the log-likelihood, AIC, BIC, and intraclass correlation coefficients (ICCs) for null models at the school level, district level, and with schools nested within districts.

|  | Log-likelihood | ICC | AIC | BIC |
| --- | --- | --- | --- | --- |
| **Bullied by someone physically** |  |  |  |  |
| School level | -2655.092 | 0.0734119 | 5314.185 | 5328.047 |
| District level | -2672.207 | 0.0162514 | 5348.414 | 5362.276 |
| School nested in District | -2652.118 | 0.0748072 | 5310.236 | 5331.029 |
| **Bullied by someone online** |  |  |  |  |
| School level | -2062.967 | 0.0812233 | 4129.934 | 4143.796 |
| District level | -2075.442 | 0.0212025 | 4154.885 | 4168.747 |
| School nested in District | -2060.536 | 0.0809087 | 4127.073 | 4147.866 |
| **Bullied someone physically** |  |  |  |  |
| School level | -3016.444 | 0.080953 | 6036.887 | 6050.749 |
| District level | -3030.893 | 0.0289737 | 6065.786 | 6079.648 |
| School nested in District | -3006.952 | 0.0841979 | 6019.905 | 6040.698 |
| **Bullied someone online** |  |  |  |  |
| School level | -2374.522 | 0.0740492 | 4753.044 | 4766.906 |
| District level | -2379.978 | 0.0260695 | 4763.955 | 4777.817 |
| School nested in District | -2366.765 | 0.0766165 | 4739.529 | 4760.322 |

Table2: shows the estimate and standard errors in the multilevel logit model for bullying outcomes, with school level, district level and school nested in district level.

|  | **β0 (SE)** | **var(district) (SE)** | **var(school) (SE)** |
| --- | --- | --- | --- |
| **Bullied by someone physically** |  |  |  |
| School level | 0.11(0.01) | - | 0.26(0.06) |
| District level | 0.13(0.01) | 0.05(0.03) | - |
| School and District level | 0.11(0.01) | 0.05(0.03) | 0.22(0.06) |
| **Bullied by someone online** |  |  |  |
| School level | 0.08(0.01) | - | 0.29(0.07) |
| District level | 0.08(0.01) | 0.07(0.04) | - |
| School and District level | 0.08(0.01) | 0.05(0.04) | 0.24(0.7) |
| **Bullied someone physically** |  |  |  |
| School level | 0.14(0.01) | - | 0.29(0.06) |
| District level | 0.16(0.02) | 0.1(0.05) | - |
| School and District level | 0.14(0.02) | 0.1(0.05) | 0.20(0.05) |
| **Bullied someone online** |  |  |  |
| School level | 0.1(0.01) | - | 0.26(0.06) |
| District level | 0.1(0.01) | 0.09(0.04) | - |
| School and District level | 0.1(0.01) | 0.09(0.05) | 0.18(0.05) |

For each bullying outcome, we fitted null multilevel logistic regression models at the school level, district level, and a three-level structure with schools nested within districts. Model comparisons using log-likelihood and AIC/BIC suggested that the school-level and school-nested-in-district models fit better than the district-only models. The intraclass correlation coefficients (ICCs) showed that clustering at the school level consistently accounted for 7–8% of the variance in bullying outcomes, whereas district-level clustering was much smaller (2–3%). Variance estimates confirmed this pattern, with school-level variance ranging from 0.22 to 0.29 compared with 0.05 to 0.10 at the district level. Given that the majority of clustering was attributable to schools and that the number of districts (n = 12) was too small for stable random-effects estimation, subsequent analyses were conducted using two-level models with children nested within schools. We have reported two multilevel models, first model included only student-level characteristics, while the second model additionally incorporated school-level characteristics.

As a robustness check, we compared results from multilevel logistic regression with those from a standard multivariate logistic regression model. Refer to Table 3 for Bullying Perpetrator outcome and Table 4 for Bullying Victimization outcome.

Adjusted odds ratios (aOR) and 95% confidence intervals (CI) from both approaches were nearly identical, indicating minimal impact of clustering. Given this consistency, results from the standard multivariate logistic regression are presented in the main text.

| Table 3: Factor associated with Bullying | | | | | | |
| --- | --- | --- | --- | --- | --- | --- |
| Characteristics | **Bullied someone physically** | | | **Bullied someone online** | | |
|  | Model 1( with student level charactristics) | Model2 (both student and school level) | Multivariate logit model (aOR) | Model 1( with student level charactristics) | Model2 (both student and school level) | Multivariate logit model (aOR) |
| **Individual level** | | | | | | |
| Gender |  |  |  |  |  |  |
| Girl | Ref | Ref | Ref | Ref | Ref | Ref |
| Boy | 1.57**[1.34,1.83] | 1.57**[1.34,1.83] | 1.57** [1.35,1.82] | 1.64**[1.37,1.96] | 1.63 **[1.36, 1.95] | 1.63** [1.37,1.94] |
| Age |  |  |  |  |  |  |
| 13-15 years | - | - | - | Ref | Ref | Ref |
| 16-17 years | - | - | - | 1.17 [0.98,1.39] | 1.17[0.98, 1.39] | 1.17 [0.98,1.39] |
| Consumption of Junk food |  |  |  |  |  |  |
| No | Ref | Ref | Ref | Ref | Ref | Ref |
| Yes | 1.34**[1.14,1.58] | 1.35**[1.15,1.59] | 1.34** [1.14,1.56] | 1.6**[1.33,1.94] | 1.61 **[1.33, 1.94] | 1.59** [1.32,1.92] |
| Skipped breakfast |  |  |  |  |  |  |
| No | Ref | Ref | Ref | Ref | Ref | Ref |
| Yes | 1.41**[1.20,1.65] | 1.4**[1.19,1.65] | 1.41** [1.20,1.65] | 1.39**[1.16,1.67] | 1.39 **[1.16, 1.67] | 1.4** [1.17,1.67] |
| Time spent on digital devise |  |  |  |  |  |  |
| <8 hr | Ref | Ref | Ref | Ref | Ref | Ref |
| >=8 hr | 1.64* [1.10,2.43] | 1.64* [1.11,2.43] | 1.67** [1.13,2.46] | 2.19**[1.47,3.26] | 2.20 **[1.48, 3.28] | 2.2** [1.49,3.25] |
| Owning a phone |  |  |  |  |  |  |
| Yes | Ref | Ref | Ref | Ref | Ref | Ref |
| No | 1.35**[1.15,1.57] | 1.34**[1.14,1.56] | 1.34** [1.15,1.57] | 2.18**[1.83,2.59] | 2.17 **[1.82, 2.58] | 2.15** [1.81,2.55] |
| Substance use |  |  |  |  |  |  |
| No | Ref | Ref | Ref | Ref | Ref | Ref |
| Yes | 1.66**[1.43,1.93] | 1.67**[1.43,1.94] | 1.68** [1.45,1.95] | 1.45**[1.22,1.73] | 1.46 **[1.22, 1.73] | 1.46** [1.23,1.73] |
| History of sexual activity |  |  |  |  |  |  |
| No | Ref | Ref | Ref | Ref | Ref | Ref |
| Yes | 1.86**[1.46,2.37] | 1.86**[1.46,2.37] | 1.84** [1.45,2.33] | 1.86**[1.43,2.41] | 1.86 **[1.43, 2.41] | 1.84** [1.43,2.38] |
| Felt depressed |  |  |  |  |  |  |
| No | Ref | Ref | Ref | Ref | Ref | Ref |
| Yes | 1.34* [1.06,1.69] | 1.33* [1.06,1.69] | 1.33*  [1.06,1.68] | 1.28 [0.99,1.66] | 1.28[0.99, 1.66] | 1.29* [1.00,1.67] |
| Felt worried |  |  |  |  |  |  |
| No | Ref | Ref | Ref | Ref | Ref | Ref |
| Yes | 1.1 [0.84,1.46] | 1.11 [0.84,1.47] | 1.1  [0.84,1.45] | 1.33 [0.99,1.79] | 1.34[1.00, 1.80] | 1.35* [1.01,1.81] |
| Felt nervous or anxious |  |  |  |  |  |  |
| No | Ref | Ref | Ref | Ref | Ref | Ref |
| Yes | 1.37* [1.04,1.80] | 1.36* [1.03,1.79] | 1.36*  [1.03,1.78] | 1.33 [0.99,1.79] | 1.33[0.99, 1.79] | 1.32  [0.99,1.77] |
| Difficulty in stay focused |  |  |  |  |  |  |
| No | Ref | Ref | Ref | Ref | Ref | Ref |
| Yes | 1.54**[1.26,1.90] | 1.54**[1.25,1.89] | 1.52** [1.24,1.86] | 1.39**[1.11,1.75] | 1.38 **[1.10, 1.74] | 1.38** [1.10,1.72] |
| Indulged in Physical fight |  |  |  |  |  |  |
| No | Ref | Ref | Ref | Ref | Ref | Ref |
| Yes | 4.68**[4.03,5.42] | 4.66**[4.03,5.40] | 4.62** [4.00,5.33] | 2.25**[1.89,2.67] | 2.24 **[1.88, 2.66] | 2.24** [1.89,2.65] |
| **Family level** | | | | | | |
| Time spent with parents |  |  |  |  |  |  |
| Never/Rarely/Sometimes | Ref | Ref | Ref | Ref | Ref | Ref |
| Most of the time/always | 0.72**[0.61,0.86] | 0.72**[0.61,0.86] | 0.73** [0.62,0.87] | 0.7**[0.58,0.85] | 0.70 **[0.58, 0.85] | 0.7** [0.58,0.85] |
| Patental supervision |  |  |  |  |  |  |
| Never/Rarely/Sometimes | Ref | Ref | Ref | Ref | Ref | Ref |
| Most of the time/always | 0.92 [0.79,1.07] | 0.92 [0.79,1.07] | 0.91  [0.78,1.05] | 1.08 [0.91,1.28] | 1.08[0.91, 1.29] | 1.07  [0.90,1.27] |
| Parental occupation |  |  |  |  |  |  |
| skilled/professional | - | - | - | Ref | Ref | Ref |
| unskilled/semi-skilled | - | - | - | 1.25* [1.02,1.53] | 1.26 * [1.03, 1.53] | 1.25* [1.02,1.52] |
| self-employed | - | - | - | 0.99 [0.78,1.27] | 0.99[0.78, 1.26] | 0.98  [0.77,1.24] |
| **School** |  |  |  |  |  |  |
| written policy/guideline/rule prohibiting bullying |  |  | - |  |  | - |
| No | - | Ref | - | - | Ref | - |
| Yes | - | 1.43 [0.87,2.34] | - | - | 0.87[0.54, 1.41] | - |
| Residence |  |  |  |  |  |  |
| Rural | - | Ref | Ref | - | Ref | Ref |
| Urban | - | 1.22 [0.95,1.59] | 1.22  [0.99,1.50] | - | 1.2[0.91, 1.58] | 1.21 [0.96,1.53] |
| var(cons) | 0.12 [0.06,0.23] | 0.11 [0.06,0.22] | - | 0.11 [0.05,0.25] | 0.11[0.04, 0.25] | - |
| ICC | 0.0355523 | 0.0326919 | - | 0.0325947 | 0.0310028 | - |
| log liklihood | -2546.442 | -2544.423 | - | -2085.165 | -2084.17 | - |
| AIC | 5124.884 | 5124.845 | - | 4208.331 | 4210.34 | - |
| BIC | 5235.781 | 5249.604 | - | 4340.02 | 4355.891 | - |
| ** p<.01, * p<.05 |  |  |  |  |  |  |

| Table 4: Factor associated with bullying victimization | | | | | | |
| --- | --- | --- | --- | --- | --- | --- |
|  | **Bullied by someone physically** | | | **Bullied by someone online** | | |
| Characteristics | Model 1( with student level charactristics) | Model2 (both student and school level) | Multivariate logit model (aOR) | Model 1( with student level charactristics) | Model2 (both student and school level) | Multivariate logit model (aOR) |
| **Individual level** | | | | | | |
| Gender |  |  |  |  |  |  |
| Girl | Ref | Ref | Ref | Ref | Ref | Ref |
| Boy | 1.91** [1.61,2.26] | 1.90 ** [1.61, 2.25] | 1.91** [1.62,2.25] | 1.4**[1.16,1.70] | 1.4**[1.15,1.70] | 1.41** [1.17,1.71] |
| Age |  |  |  |  |  |  |
| 13-15 years | - | - | - | Ref | Ref | Ref |
| 16-17 years | - | - | - | 1.13 [0.93,1.36] | 1.12 [0.93,1.35] | 1.12 [0.93,1.35] |
| Consumption of Junk food |  |  |  |  |  |  |
| No | Ref | Ref | Ref | Ref | Ref | Ref |
| Yes | 1.15 [0.97,1.36] | 1.16 [0.98, 1.37] | 1.16 [0.98,1.37] | 1.24* [1.01,1.52] | 1.25* [1.02,1.52] | 1.25* [1.02,1.52] |
| Skipped breakfast |  |  |  |  |  |  |
| No | Ref | Ref | Ref | Ref | Ref | Ref |
| Yes | 1.33** [1.12,1.58] | 1.33 ** [1.12, 1.58] | 1.33** [1.12,1.58] | 1.46**[1.20,1.77] | 1.46**[1.20,1.77] | 1.47** [1.21,1.78] |
| Time spent on digital devise |  |  |  |  |  |  |
| <8 hr | Ref | Ref | Ref | Ref | Ref | Ref |
| >=8 hr | 0.7 [0.44,1.13] | 0.71[0.44, 1.13] | 0.69 [0.43,1.11] | 1.15 [0.72,1.85] | 1.16 [0.72,1.86] | 1.11 [0.70,1.77] |
| Feeling unsafe while going to school |  |  |  |  |  |  |
| No | Ref | Ref | Ref | Ref | Ref | Ref |
| Yes | 1.94** [1.64,2.29] | 1.93 ** [1.64, 2.28] | 1.93** [1.64,2.27] | 1.86**[1.53,2.26] | 1.86**[1.53,2.25] | 1.83** [1.51,2.21] |
| Owning a phone |  |  |  |  |  |  |
| Yes | Ref | Ref | Ref | Ref | Ref | Ref |
| No | 1.22* [1.04,1.44] | 1.21 * [1.02, 1.43] | 1.22* [1.03,1.44] | 1.88**[1.56,2.27] | 1.87**[1.55,2.26] | 1.87** [1.55,2.25] |
| Substance use |  |  |  |  |  |  |
| No | Ref | Ref | Ref | Ref | Ref | Ref |
| Yes | 1.37** [1.16,1.61] | 1.38 ** [1.17, 1.62] | 1.38** [1.17,1.63] | 1.18 [0.97,1.43] | 1.19 [0.98,1.44] | 1.2  [0.99,1.45] |
| History of sexual activity |  |  |  |  |  |  |
| No | Ref | Ref | Ref | Ref | Ref | Ref |
| Yes | 1.55** [1.21,2.01] | 1.56 ** [1.21, 2.01] | 1.53** [1.19,1.96] | 1.87**[1.42,2.46] | 1.87**[1.42,2.47] | 1.85** [1.41,2.42] |
| Felt depressed |  |  |  |  |  |  |
| No | Ref | Ref | Ref | Ref | Ref | Ref |
| Yes | 1.44** [1.13,1.83] | 1.43 ** [1.12, 1.82] | 1.42** [1.12,1.80] | 1.5**[1.15,1.96] | 1.5**[1.14,1.96] | 1.48** [1.14,1.93] |
| Felt worried |  |  |  |  |  |  |
| No | Ref | Ref | Ref | Ref | Ref | Ref |
| Yes | 1.29 [0.97,1.71] | 1.3 [0.98, 1.73] | 1.3 [0.98,1.72] | 1.2 [0.87,1.64] | 1.2 [0.88,1.65] | 1.2 [0.88,1.64] |
| Felt nervous or anxious |  |  |  |  |  |  |
| No | Ref | Ref | Ref | Ref | Ref | Ref |
| Yes | 1.26 [0.94,1.67] | 1.25[0.94, 1.66] | 1.25 [0.94,1.66] | 1.33 [0.97,1.82] | 1.32 [0.97,1.81] | 1.34 [0.99,1.82] |
| Difficulty in stay focused |  |  |  |  |  |  |
| No | Ref | Ref | Ref | Ref | Ref | Ref |
| Yes | 1.61** [1.31,1.99] | 1.60 ** [1.30, 1.98] | 1.61** [1.31,1.98] | 1.53**[1.20,1.95] | 1.52**[1.20,1.94] | 1.52** [1.20,1.92] |
| Indulged in Physical fight |  |  |  |  |  |  |
| No | Ref | Ref | Ref | Ref | Ref | Ref |
| Yes | 3.04** [2.60,3.56] | 3.03 ** [2.59, 3.55] | 3.04** [2.60,3.56] | 2.27**[1.88,2.73] | 2.26**[1.87,2.72] | 2.26** [1.88,2.71] |
| **Family level** | | | | | | |
| Time spent with parents |  |  |  |  |  |  |
| Never/Rarely/Sometimes | Ref | Ref | Ref | Ref | Ref | Ref |
| Most of the time/always | 0.66** [0.55,0.79] | 0.66 ** [0.55, 0.78] | 0.65** [0.55,0.78] | 0.67**[0.54,0.82] | 0.67**[0.54,0.82] | 0.67** [0.55,0.82] |
| Patental supervision |  |  |  |  |  |  |
| Never/Rarely/Sometimes | Ref | Ref | Ref | Ref | Ref | Ref |
| Most of the time/always | 1.01 [0.86,1.19] | 1.02[0.87, 1.19] | 1.02 [0.87,1.19] | 1.03 [0.85,1.24] | 1.03 [0.85,1.24] | 1.02  [0.85,1.22] |
| Parental occupation | - | - | - | - | - | - |
| unskilled/semi-skilled | - | - | - | - | - | - |
| skilled/professional | - | - | - | - | - | - |
| self-employed | - | - | - | - | - | - |
| **School level** | | | | | | |
| written policy/guideline/rule prohibiting bullying |  |  |  |  |  |  |
| No | - | Ref | - | - | Ref | - |
| Yes | - | 1.16[0.74, 1.82] | - | - | 1.2 [0.67,2.13] | - |
| Residence |  |  |  |  |  |  |
| Rural | - | Ref | Ref | - | Ref | Ref |
| Urban | - | 1.35 *[1.06, 1.73] | 1.35** [1.09,1.67] | - | 1.26 [0.93,1.70] | 1.27 [0.99,1.63] |
| ICC | 0.0210248 | 0.0170857 | - | 0.0414437 | 0.037561 | - |
| log liklihood | -2305.708 | -2302.695 | - | -1818.962 | -1817.748 | - |
| AIC | 4645.415 | 4643.39 | - | 3673.924 | 3675.497 | - |
| BIC | 4763.243 | 4775.08 | - | 3798.682 | 3814.117 | - |
| ** p<.01, * p<.05 |  |  |  |  |  |  |
